# Thermal Gas Flow Sensor Using SiGe HBT Oscillators Based on GaN/Si SAW Resonators

**DOI:** 10.3390/mi16101151

**Published:** 2025-10-10

**Authors:** Wenpu Cui, Jie Cui, Wenchao Zhang, Guofang Yu, Di Zhao, Jingqing Du, Zhen Li, Jun Fu, Tianling Ren

**Affiliations:** 1 School of Integrated Circuits and Beijing National Research Center for Information Science and Technology (BNRist), Tsinghua University, Beijing 100084, China; cuiwenpu@163.com (W.C.); cui_jie@tsinghua.edu.cn (J.C.); 17786779852@163.com (W.Z.); yugf24@nudt.edu.cn (G.Y.); 2 College of Computer Science and Technology, National University of Defense Technology, Changsha 410073, China; 3 Beijing Sevenstar Flow Co., Ltd., Beijing 100176, China; zhaodi@sevenstar.com.cn (D.Z.); dujingqing@sevenstar.com.cn (J.D.)

**Keywords:** SAW, thermal, GaN, gas flow sensor, SiGe HBT

## Abstract

This paper presents a thermal gas flow sensing system, from surface acoustic wave (SAW) temperature sensor to oscillation circuit and multi-module miniaturization integration. A single-port GaN/Si SAW resonator with single resonant mode and excellent characteristics was fabricated. Combined with an in-house-developed SiGe HBT, a temperature-sensitive high-frequency oscillator was constructed. Under constant temperature control, system-level flow measurement was achieved through dual-oscillation configuration and modular integration. The fabricated SAW device shows a temperature coefficient of frequency (TCF) −28.29 ppm/K and temperature linearity 0.998. The oscillator operates at 1.91 GHz with phase noise of −97.72/−118.62 dBc/Hz at 10/100 kHz offsets. The system demonstrates excellent dynamic response and repeatability, directly measuring 0–50 sccm flows. For higher flows (>50 sccm), a shunt technique extends the test range based on the 0–10 sccm linear region, where response time is <1 s with error <0.9%. Non-contact operation ensures high stability and long lifespan. The sensor shows outstanding performance and broad application prospects in flow measurement.

## 1. Introduction

The test and control of fluid flow require accurate flow measurement technology in key areas such as medical gas management, semiconductor equipment manufacturing, photovoltaic industry, industrial control and environmental monitoring [1,2,3,4]. There are many ways to measure flow, such as thermal flowmeters, variable cross-section flowmeters, ultrasonic flowmeters and mechanical flowmeters [5,6,7]. With the wide application and rapid development of flowmeters in many fields, there are more requirements for precision control of flow, miniaturization of equipment structure, control of corrosive or toxic gases and lower costs.

Thermal flowmeters are usually the main method to measure gas flow. With a heating source heating an object in contact with flowing gas, the thermal flowmeter measures the heat transferred by the gas [8]. As a kind of mechanical wave, SAW propagates on the surface of a piezoelectric substrate at a depth of one to two wavelengths. Because the electrical properties of surface wave devices are easily affected by chemical or physical disturbances on the surface of the substrate, detection experiments can be carried out according to changes in related chemical or physical quantities. SAW devices can realize the conversion of acoustic waves and electrical signals [9], and are sensitive to temperature changes. When the temperature changes, the sound velocity and stress state of the piezoelectric material of the SAW device change, resulting in drift of the resonant frequency of the SAW. Therefore, temperature and flow change can be calculated by measuring frequency drift. In addition, SAW-based sensors have the advantages of strong stability, high sensitivity, high resolution, small size, low power consumption and low cost, which have attracted more and more attention.

There are many piezoelectric materials used to fabricate SAW devices, such as lithium niobate, lithium tantalate, quartz and zinc oxide [10,11,12]. However, these traditional piezoelectric materials used to prepare SAW devices have bottlenecks such as low frequency and difficulty in monolithic integration. As a wide-bandgap semiconductor, GaN has the advantages of high-breakdown electric field, high electron mobility and high temperature tolerance, and plays an important role in the field of RF and power electronics [13,14,15]. In addition, GaN also has excellent piezoelectric properties, making it an important choice for the preparation of SAW devices [16,17,18,19], which can also be monolithically integrated with active devices such as high-electron-mobility transistors. Through integration, an intelligent sensing system integrating acoustic sensing and electrical signal processing functions was constructed [20,21,22,23].

Though some studies have carried out research on SAW resonator gas flowmeters, they were equipped with large and expensive vector network analyzers and only achieved device-level detection [24,25]. In addition, in some studies, the SAW resonator was in direct contact with the measured gas. If the measured gas is toxic or has strong corrosivity, the stability and service life of the SAW resonator [26,27,28] will be seriously affected. In many other studies, the SAW devices were delay lines made of traditional piezoelectric materials, and the operating frequency was at the MHz level [29,30], limiting the development and application of the gas flow sensor in practice.

In our research, a thermal gas flow sensor using SiGe HBT oscillators based on GaN/Si SAW resonators was successfully developed. The sensing system included a GaN/Si SAW resonator (self-developed), SAW/SiGe HBT oscillator (self-developed), mixer, intermediate frequency signal processing circuit, digital frequency meter based on a field-programmable gate array (FPGA), constant temperature controller and heater, etc. The thermal gas flow sensor heats the ventilation pipeline to a constant temperature, which is maintained through a heater equipped with a constant temperature controller. When the measured gas flows past the upstream SAW resonator, it will take away the heat from the upstream SAW position and reduce its temperature. The heat taken from upstream and the heater will be transferred to the downstream SAW position, resulting in an increase in the downstream SAW temperature. As a result, the temperature difference between the upstream and downstream GaN/Si SAW resonators leads to a resonance frequency difference and thus to an oscillation frequency difference between the upstream and downstream SAW-based SiGe HBT oscillators. The upstream and downstream RF signals are then mixed and down-converted to intermediate frequency signals by the mixer. The intermediate frequency signal processing circuits are used to amplify, shape and transform the sinusoidal intermediate frequency signals into a square wave signal. Finally, the digital frequency meter based on FPGA is used to count the frequency of the intermediate frequency square wave signal and detect the gas flow. Through the design and implementation of the above flow-test module, oscillation circuit and signal processing module, a complete measurement system was developed and demonstrated as a thermal gas flow sensor based on SiGe HBT oscillators and with GaN/Si SAW resonators. The system realizes miniaturization, integration, portability and mobility. The detection range, sensitivity, accuracy, repeatability, stability and response time of the flow sensor were experimentally tested and characterized, proving its excellent performance as a high integration solution for accurate and rapid detection of gas flow.

## 2. Design and Experiment

### 2.1. Process, Design and Characterization of GaN/Si SAW Sensors

The epitaxial layer of the SAW device is a compound, thin-film single crystal prepared by metal organic chemical vapor deposition (MOCVD) on a high-resistance silicon (111) substrate. First, a 20 nm AlN nucleation layer was grown at 780 °C followed by a 100 nm AlN film grown at 1100 °C. Then, a 1.8 μm graded Al_x_Ga _(1-x)_N buffer layer was grown at 990 °C. Finally, three successive growths of 700 nm undoped GaN film at 1050 °C, 1 μm carbon-doped GaN film at 970 °C, and 1.5 μm undoped GaN film at 1050 °C completed the epitaxial process. By using electron beam evaporation, a 10 nm titanium metal film and a 100 nm aluminum metal film were deposited as adhesion layer and electrode layer, respectively, on the substrate [31]. Metal lift-off technology was used for patterning the metal electrodes.

**Figure 1:**
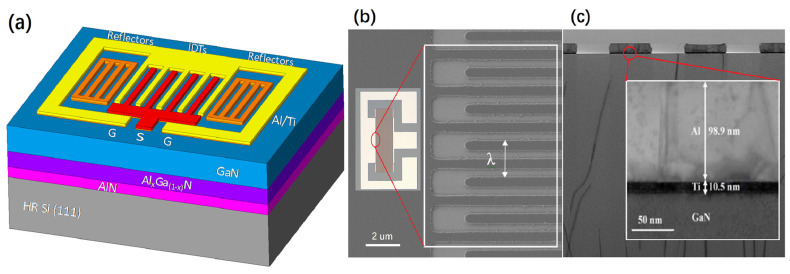
(**a**) Simplified schematic diagram of a single-port resonator based on a GaN/Al_x_Ga _(1-x)_ N/AlN/Si (111) multilayer structure. (**b**) SEM image of a GaN/Si SAW resonator with a λ of 2.0 µm. (**c**) TEM image of a cross-section through SAW IDTs.

The single-port GaN/Si SAW resonator were layout designed for interdigital transducer (IDT) and reflection grating as shown in Figure 1a. The wavelength (λ) is 2 μm, the number of IDTs (N_IDT_) is 250 pairs and the number of the reflection grating (N_GR_) on both sides is 20 pairs. The width and space of the metal strips of the reflection grating are the same as those of the IDT. The acoustic aperture is 40 λ, the width of the interdigital metal electrode is λ/4, the center distance between the metal electrodes is λ/2, the distance between the end of the metal electrode and the bus bar is λ and the distance between the reflection grating and the IDT is λ/4. The metallization ratio represents the ratio between the width of the metal interdigital electrode and the center distance of the adjacent electrodes. According to the calculation of the structural parameters, the metallization ratio is 0.5. The optical microscope and scanning electron microscope (SEM) images of the GaN Si SAW device structure with a single port are shown in Figure 1b, and the transmission electron microscope (TEM) images are shown in Figure 1c.

### 2.2. Design of SiGe HBT High-Frequency Oscillator Based on GaN/Si SAW Resonator

A Heterojunction Bipolar Transistor (HBT) is a high-speed transistor that utilizes different semiconductor materials (such as GaAs, InP or SiGe) to form junctions. Thanks to its heterostructure, the HBT can operate at extremely high frequencies with excellent performance. An HBT oscillator refers to a high-frequency signal generation circuit that uses the HBT transistor as the core active component for amplification and feedback. When gas flows through a pipeline, the temperatures of the upstream and downstream SAW resonators change. As a passive component, an SAW resonator cannot independently output its resonant frequency. Therefore, in the HBT oscillator circuit, the HBT acts as the active element while the SAW resonator serves as the feedback component, working together to generate a stable high-frequency AC signal at the resonant frequency.

**Figure 2:**
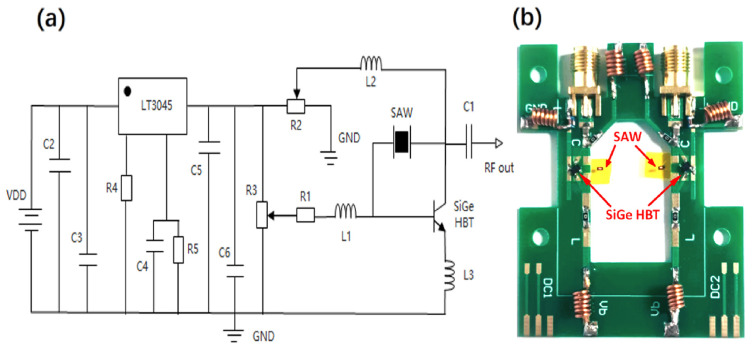
(**a**) The schematic diagram of a high-frequency SiGe HBT oscillation circuit based on a GaN SAW resonator. (**b**) Physical display of upstream and downstream oscillation circuits with a working frequency of 1.91 GHz.

The high-frequency oscillator was designed and implemented based on the single-port GaN/Si SAW resonator as a feedback element and an SiGe HBT device as an active oscillating transistor. According to the circuit schematic (Figure 2a) of the oscillator, the active device adopts the high-frequency SiGe HBT KT9041 independently developed by our group. The oscillator applies a bias voltage of 2 V between the collector (C) and the emitter (E). By adjusting the bias voltage of the base (B), the HBT operates at a collector current of 25 mA, with the cut-off frequency (f_T_) being 25 GHz under this bias condition. According to the circuit diagram, the GaN/Si SAW resonator is connected between the collector C and the base B of the HBT, thus forming a positive feedback circuit for initiating and maintaining the oscillation. The three electrodes of the HBT are connected with a high-frequency inductor, which is used for stabilizing the current, circuit tuning, filtering and so on. The high-performance linear regulator step-down chip LT3045 has an output ripple of better than 1 mV, a load regulation rate of ±0.5%, and an extremely low output noise. The DC voltage is reduced to 1.2 V by a linear voltage-stabilized step-down circuit, and different voltages are applied to the collector and the base by adjusting two adjustable potentiometers, R2 and R3, respectively. The physical display of the oscillator is shown in Figure 2b. The GaN/Si SAW resonator and SiGe HBT were interconnected by wire bonding on the PCB, constructing the high-frequency oscillator.

### 2.3. Thermal Simulation and Temperature Controller Design

**Figure 3:**
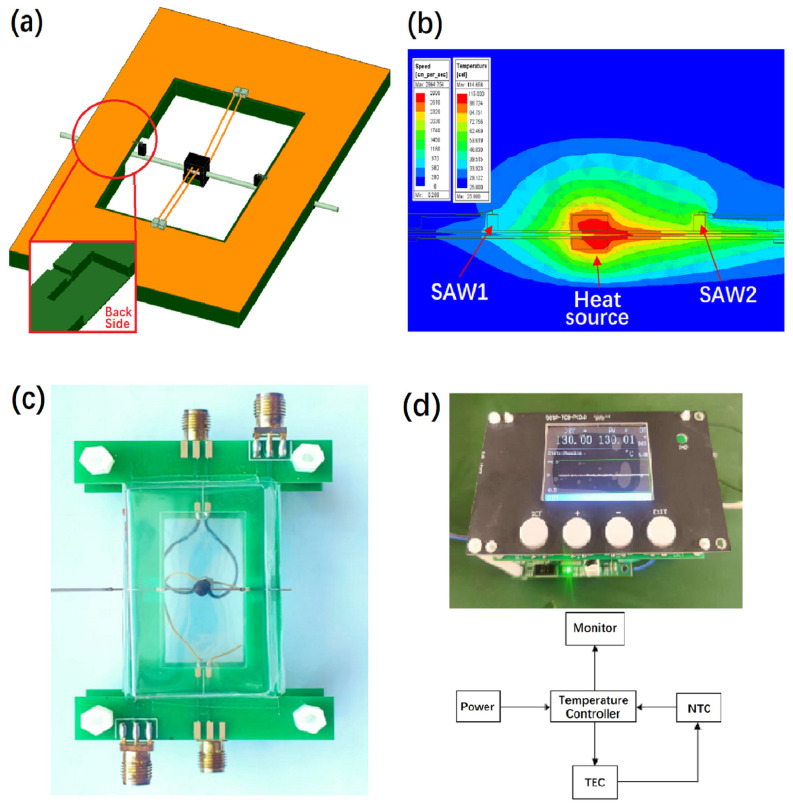
(**a**) The establishment of a three-dimensional simulation structure model with fully coupled thermal field and airflow. (**b**) Thermal field distribution of upstream and downstream SAW resonators under constant heating source and ventilation flow state. (**c**) Physical display of PCB special design and upstream and downstream SAW resonators located 7–8 mm away from the heating source. (**d**) The principle and implementation of a temperature controller.

The purpose of gas thermal simulation is mainly to verify the principle, determine the placement position of two GaN/Si SAW resonators and minimize the influence of the thermal load of a printed circuit board (PCB) as a heat source, so as to better realize gas flow detection. Using ANSYS Icepak thermal simulation software (Version 2023 R1), a three-dimensional model of thermal-flow coupling was established and multiple gas thermal simulations were performed. The optimal position of the two resonators and the optimal structure of the PCB were determined by scanning different parameters defined by ANSYS. The structure distribution is shown in Figure 3a. As for the gas pipeline made of material 316 L with corrosion resistance, the outer diameter is 0.5 mm, the inner diameter is 0.2 mm and the length is greater than 50 mm. The thermostat was fixed at the center of the vent pipe and the placement position of SAW1 is 7–8 mm away from the upstream of the thermostat. Axisymmetric with it, 7–8 mm from the downstream position of the thermostat gives the SAW2 placement position. Nitrogen with a flow rate of 0–70 standard cubic centimeters per minute (sccm) was introduced into the pipeline with the temperature difference between the upstream and downstream GaN/Si SAW resonators being ΔT = T_SAW2_ − T_SAW1_. The distribution of upstream and downstream temperature fields can be seen in Figure 3b. As the carrier board of the oscillation circuit and ventilation pipeline, the PCB has also become the thermal load of the thermostat. It is necessary to design the PCB specially. The hollow size of the central part of the PCB was optimized to be 20 mm × 17 mm × 1.6 mm, meanwhile setting two grooves and two steps on its back. Grooves with a size of 3 mm × 0.8 mm × 0.8 mm were used to fix the ventilation pipe, and the step size was set at 9 mm × 4 mm × 1.4 mm. The position of the SAW devices and the special PCB design are shown in Figure 3c. The optimization of the PCB succeeded in reducing the thermal load effect and improving the sensitivity of gas flow detection.

The temperature controller is an integrated device to achieve high precision temperature control, mainly including semiconductor PID digital controller, digital display, temperature sensor, and heating wire. The temperature controller achieves high-precision temperature rise and fall in a very short time, and maintains a constant temperature with a temperature accuracy of ±0.01 °C. PT100 was adopted as the temperature sensor with a temperature tolerance more than 300 °C. Enameled nickel–chromium wire was chosen as the heating wire with a diameter of 30 μm. After multiple winding in the center of the ventilation pipe, the resistance of the heating wire was adjusted to 80–130 Ω. At the center of the heating wire, a temperature sensor was placed to stick to the heating wire and the ventilation pipe with the input and output ends of the electrode extracted and packaged to form a thermostat of 2 mm × 2 mm × 2 mm. The principle and physical display of the temperature controller are shown in Figure 3d.

### 2.4. System Design

**Figure 4:**
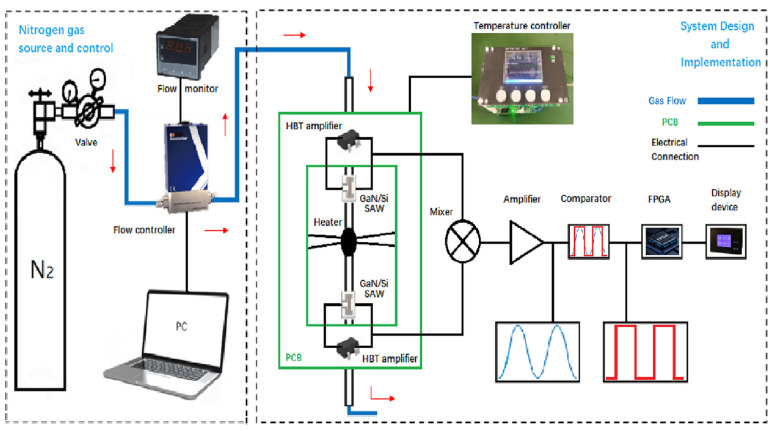
Complete system design diagram of thermal gas flow sensor using SiGe HBT oscillators based on GaN/Si SAW resonator.

The system design of the thermal gas flow sensor using SiGe HBT oscillators based on GaN/Si SAW resonators is shown in Figure 4. The gas source used in the experiment is compressed nitrogen at a constant temperature (about 25 °C). In order to ensure the stability of the airflow and reduce the interference caused by the flow fluctuation, a long gas pipeline was employed in the experiment. The gas temperature at the outlet of the gas pipe was measured by a pipe thermometer to ensure the validity of the experimental data. The flow controller (CS300 series) was used to control the flow of the test gas, enabling the flow value to be read by the flow meter in real time.

The output of the SAW sensor is the change in working frequency, so it is very important to realize the output and processing of the frequency signal. The temperature controller of the experimental design was used as a high-precision constant-temperature heating source for the system. When different flow rates of gas pass through the gas pipeline, the upstream GaN/Si SAW resonator is in the process of cooling. Since the heat source thermostat heats the gas, the gas carries part of the heat to the downstream GaN/Si SAW resonator, and the downstream GaN/Si SAW resonator is in the process of heating. As a result, there is a temperature difference between the upstream GaN/Si SAW resonator and the downstream one. In addition, the GaN/Si SAW resonator has excellent wide-temperature resonance, low loss and high Q value, and can directly output frequency signals without analog-to-digital conversion. The SiGe HBT high-frequency oscillator based on the single-port GaN/Si SAW resonator was employed to measure the frequency response of the upstream and downstream resonators. In the experiment, the double oscillator technology was used to eliminate the influence of ambient temperature change on the thermal gas flow sensor. The outputs of the upstream and downstream oscillators were taken as the input and local oscillation ports, respectively, of the mixer. The mixer outputs a MHz-level intermediate frequency signal, which is the difference between the output frequencies of the upstream and downstream SAW resonators due to the different resonant temperatures. Because the GaN/Si SAW resonator has a high Q value and a single resonant mode, the mixer with a suitable frequency band can be used to amplify the intermediate frequency signal directly without a filter. The amplified signal was output to the comparator for converting the sinusoidal signal into a square wave signal. Then the square wave signal was input into the FPGA used to program and calculated to obtain the corresponding gas flow data and display them on the meter screen. The system successfully integrated the functions of mixing, shaping, frequency division and counting and final detection of gas flow.

## 3. Results and Discussion

In order to ensure that the thermal gas flow sensor can work with high precision and high stability, the experiment carried out module tests and system tests on each module of the complete system step by step.

**Figure 5:**
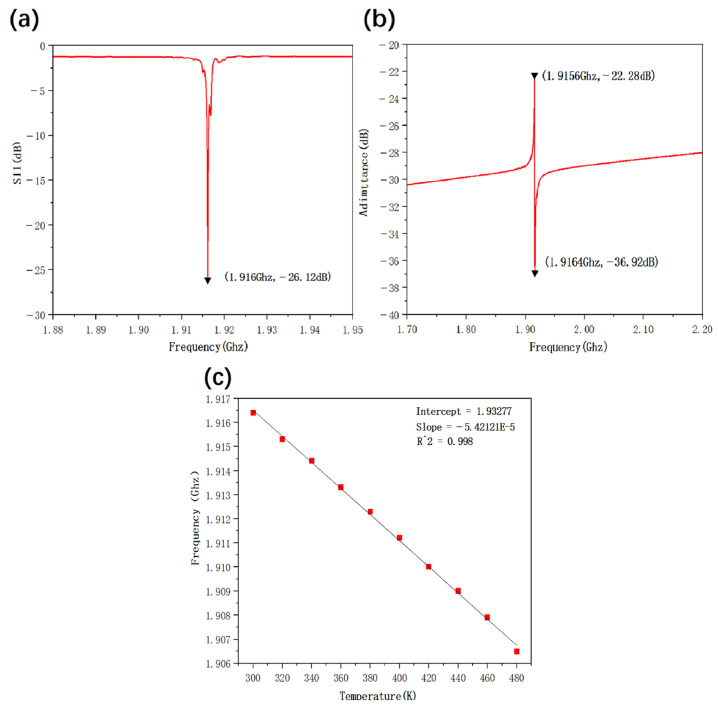
(**a**) S_11_ parameters of GaN/Si SAW resonator. (**b**) Admittance response of GaN/Si SAW. (**c**) Variation of GaN/Si SAW resonant frequency with temperature from 300 K to 480 K.

Firstly, the S-parameter measurement was completed for the single-port GaN/Si SAW resonator. As shown in Figure 5a, the S_11_ of the GaN/Si SAW resonator reaches −26 dB at the resonant frequency of 1.916 GHz, demonstrating good resonance characteristics. Meanwhile, the S_11_ exhibits a large reflection at other frequency points except the resonant frequency, indicating a single resonant mode of the resonator. The measured S parameters were converted into admittance parameters [32], through the conversion formula as follows:

(1) $$Y_{11}=\frac{1}{Z_{0}}\frac{1-S_{11}}{1+S_{11}}$$

With characteristic impedance Z_0_ of 50 Ω, as shown in Figure 5b, the converted admittance characteristics show the series and parallel resonance frequencies. The temperature dependence of GaN/Si SAW resonators was also tested in the temperature range of 300 K to 480 K. The frequency variation of the GaN/Si SAW resonator with temperature is shown in Figure 5c. The results show that as the temperature increases, the resonant frequency of the SAW device decreases in a good linear relationship. The goodness of fit R^2^ is 0.998, and the temperature change rate is −54.212 kHz/K. Temperature coefficient of frequency (TCF) can be used to quantitatively describe this temperature dependent frequency drift. TCF is a key parameter to measure the temperature sensitivity of SAW devices [33], as defined in (2) below:

(2) $$TCF=\frac{1}{ƒ\left(T_{0}\right)}\frac{\partial ƒ}{\partial T}$$

where T_0_ is the reference temperature normally set at 300 K and $ƒ\left(T_{0}\right)$ is the resonant frequency at the reference temperature. The TCF of the GaN/Si SAW resonator was measured to be −28.29 ppm/K. Compared with other piezoelectric materials, although the TCF of the GaN/Si SAW resonator is not high, it demonstrated a single resonant mode, good resonant characteristics, and highly linear frequency dependence on temperature in the temperature range of 300 K to 480 K.

**Figure 6:**
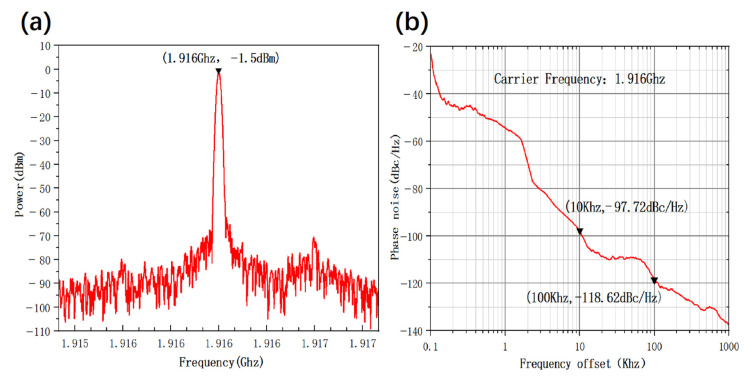
Room temperature testing of oscillation circuit based on GaN/Si SAW resonator and SiGe HBT: (**a**) Spectrum diagram of oscillating circuit. (**b**) Analysis of phase noise in oscillatory circuits.

Then, based on the GaN/Si SAW resonator, the SiGe HBT high-frequency oscillator was designed, constructed and tested by the spectrum analyzer (N9010B). In the experiment, the oscillation circuit was protected with an electromagnetic shielding cover against various electromagnetic interferences such as external environment and power voltage ripple. This shielding cover was also used to isolate the external interferences of ambient temperature and air flow. A step-down circuit with low ripple and noise was employed as the voltage source module. By using our self-developed high-frequency SiGe HBT as the active device, the oscillator achieved high performance and lower power consumption in operations where Vc was 0.75 V, Vb was 0.85 V and Ic was 7 mA. At this time, as shown in Figure 6a, the oscillation frequency was 1.916 GHz and the output power could reach −1.5 dBm. Good phase noise characteristics are shown in Figure 6b. The output power and output frequency of the oscillator can be adjusted by tuning the Vc and Vb voltage. It should be emphasized that the low power consumption of the oscillator managed to guarantee the proper and stable operation of the sensing system. In addition, the oscillator was fixed in a closed cavity with balanced thermal field and constant temperature, effectively preventing the external air flow from interfering with the internal environment.

**Figure 7:**
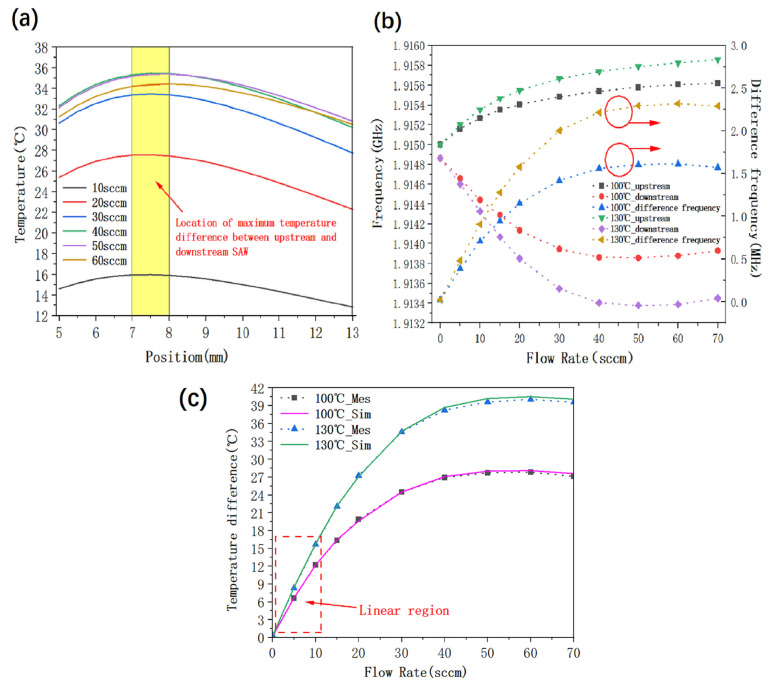
(**a**) Thermal flow simulation results: variation in temperature difference between upstream and downstream of GaN/Si SAW resonator with position distribution under different airflows. (**b**) Test results of constant heat source at 100 °C and 130 °C: frequency response of upstream and downstream GaN/Si SAW resonators with airflow variation. (**c**) Comparison of simulation and test results with constant heat sources of 100 °C and 130 °C: Response of temperature difference between upstream and downstream GaN/Si SAW resonators to changes in airflow.

Regarding the GaN/Si SAW resonators and the SiGe HBT oscillators in the upstream and downstream, respectively, of the thermal gas flow sensor system, the first problem to be solved is the most suitable distances from the thermostat. The experiment used Ansys Icepak simulation software to establish the airflow thermal simulation model. Simulation result 1, shown in Figure 7, indicates that the specific position of the maximum temperature difference between the upstream and downstream GaN/Si SAW resonators basically does not change with the size of the airflow. For a vent pipe made of 316 L material, the maximum temperature difference between the upstream and downstream GaN/Si SAW resonators corresponds to a specific position of 7–8 mm. Simulation result 2 shows that when the thermostat heats the gas pipeline at a constant temperature, a part of the heat is transmitted to the PCB carrier plate, which directly leads to a sharp decrease in the temperature difference between the upstream and downstream GaN/Si SAW resonators, and the measurement effect becomes worse. After careful simulation and optimization, the specific PCB structure was finalized as shown in the above thermal simulation design. The designed PCB structure was then processed and tested as shown in Figure 7b where the test data under temperatures of 100 °C and 130 °C are compared. The test results show that the higher the temperature, the more obvious the temperature difference between the upstream and downstream GaN/Si SAW resonators, and the more conducive to gas flow control. However, considering the actual situation, the heating temperature for testing some gases should not be too high, otherwise the chemical properties of the tested gas will change. Therefore, the heating temperature of the system should be set according to the actual situation of the tested gas. The frequency differences measured by the test data were converted into actual temperature differences with the aid of the TCF of the GaN/Si SAW resonator. As shown in Figure 7c, the measured temperature differences are in good agreement with the corresponding simulation data. The system can directly and accurately measure small gas flow rates in the 0–50 sccm range. When measuring gas flow rates >50 sccm, using the linear range of 0–10 sccm, through gas diversion technology, such as installing a small diameter measuring branch diversion device next to the main pipeline and measuring the proportional relationship between branch flow and total flow, the total flow rate can be calculated. When the temperature is 130 °C, the flow rate was found to have good linearity in the range of 0–10 sccm. Therefore, the relationship between the flow rate (FR) and the temperature difference ΔT in the range of 0–10 sccm can be obtained by linear fitting as follows:

(3) FR=0.653·ΔT−0.067

where FR is the measured gas flow rate and the slope and intercept were obtained by fitting the relationship between the measured temperature difference ΔT and the flow rate through the FPGA.

For the thermal gas flow sensor, the temperature control needs to be fast and stable with high precision and electromagnetic interference tolerance. The traditional PID controller is unable to meet the demand. The system used a semiconductor PID controller, with efficient and accurate control performance and less electromagnetic interference, as the core component of the closed-loop control system to provide accurate and stable constant temperature for the system.

**Figure 8:**
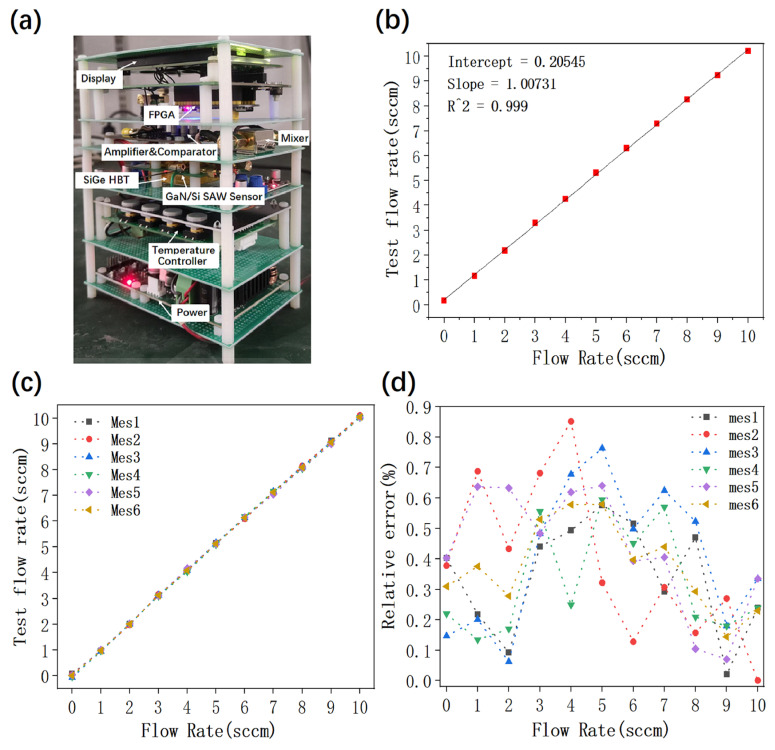
Complete system testing of thermal gas flow sensor using SiGe HBT oscillators based on GaN/Si SAW resonators. (**a**) Physical display and distribution of positions of various modules in the system. (**b**) Comparison between the gas flow rate tested by this system and the flow rate of a standard flowmeter. (**c**) Repetitive testing of this system. (**d**) Relative error distribution of repeatability testing in this system.

Finally, according to the design principle of the thermal gas flow sensor, all of the above function modules were integrated into a demo system in the experiment. A photograph of the experimental system sample is shown in Figure 8a. The overall system size is 8 cm × 10 cm × 12 cm, consisting altogether of five layers from bottom to top as shown in the picture. The first layer is the power supply module. Through the AC 220 V to DC 12 V converter, the 12 V DC voltage is directly output to the system for the power supply. The second layer is the temperature control module. The third layer is the core layer, including the high-frequency oscillator composed of SiGe HBT and GaN/Si SAW resonator, vent pipe and thermostat. The fourth layer is the amplifier, comparator and mixer. After calculating the frequency difference for the upstream and downstream RF signals, the sine wave signal is converted into a square wave signal. The fifth layer is FPGA and display. The signal is processed by frequency division, the formula after fitting the test data is programmed into FPGA and the measured information flow is transmitted to the display screen.

According to the flow range of the linear region of the system, the large gas flow rate of >50 sccm can be measured through gas diversion technology. Therefore, multiple nitrogen ventilation tests were carried out in the flow range of the linear region of the complete system. Taking a standard gas flowmeter for calibration, the relationship between the flow measured by our thermal gas flow sensor and the calibration flow rate was calculated with FPGA. As shown in Figure 8b, the equation can be obtained by linear fitting:

(4) y=1.00731x+0.02545

It can be seen that the slope is almost equal to 1, which means that the thermal gas flow sensor has excellent linearity (R^2^ = 0.999). As shown in Figure 8c, the entire system has a high degree of repeatability and the system response time is less than 1 s. By comparing the flow rates measured by this system and the standard flowmeter as shown in Figure 8d, the relative error is less than 0.9%. Our thermal gas flow sensor shows extremely high measurement accuracy.

Table 1 shows a characteristic comparison with other SAW gas flow sensors. Compared to other SAW sensors, our thermal gas flow sensor completely eliminates the reliance on large, expensive instruments such as network analyzers, requires no contact with the measured gas and significantly improves the sensor’s service life and stability. The operating frequency of our system is 1.916 GHz, which provides high test sensitivity—far exceeding the operating frequencies reported in other studies. The system enables direct detection of micro-flow rates in the range of 0–50 sccm. Larger flow rates than 50 sccm can be measured by utilizing the linear region of 0–10 sccm in this system combined with flow-splitting technology. The excellent performance of this sensor contributes to expanding the applications of SAW gas flow sensors.

## 4. Conclusions

In summary, this study employed GaN piezoelectric materials to develop a GaN/Si SAW resonator on a silicon substrate, which exhibits a single resonance mode at 1.916 GHz with favorable resonant characteristics. A single-port GaN/Si SAW resonator was integrated with a research group-developed SiGe HBT to construct a high-frequency oscillator characterized by structural simplicity, low component count, low power consumption, and excellent phase noise performance. A dual-oscillator configuration was adopted to compensate for ambient temperature variations in the thermal gas flow sensor. A three-dimensional thermal fluid-coupled model was established using ANSYS Icepak to optimize the upstream and downstream placement of the SAW resonators and help design a specialized PCB layout and structure. Based on the linear temperature–frequency relationship of the GaN/Si SAW resonator, the system managed to measure resonant frequency shifts under constant temperature and thus flow rate using separate upstream and downstream oscillators. System-level flow sensing was achieved through integrated signal processing components including mixers, amplifiers, comparators, an FPGA and a display unit. The thermal flow sensor demonstrates high measurement accuracy, excellent stability and a response time of less than 1 s. It enables direct micro-flow detection in the 0–50 sccm range without requiring external network analyzers. By utilizing the linear range of 0–10 sccm combined with flow-splitting technology, the system achieves measurement capabilities beyond 50 sccm. The non-contact sensing mechanism significantly enhances operational lifetime and stability. Its compact design, portability and high performance indicate strong potential for practical applications in gas flow measurement.

## Figures and Tables

**Table 1:** Summary of the characteristics of different SAW gas flow sensors.

System	Frequency (MHz)	Contact with Gas	Dependency on Network Analyzer	Flow Range (sccm)	Reference
No	24.5	Yes	Yes	0–1000	[24]
No	124	Yes	Yes	-	[25]
Yes	38.2	Yes	Yes	0–1000	[27]
Yes	1916	No	No	0–50	This Work

## Data Availability

The data presented in this article are available on reasonable request from the corresponding authors.
